# Medical imaging deep learning with differential privacy

**DOI:** 10.1038/s41598-021-93030-0

**Published:** 2021-06-29

**Authors:** Alexander Ziller, Dmitrii Usynin, Rickmer Braren, Marcus Makowski, Daniel Rueckert, Georgios Kaissis

**Affiliations:** 1 Institute for Diagnostic and Interventional Radiology, Klinikum rechts der Isar, School of Medicine, Technical University of Munich, Munich, Germany; 2 Artificial Intelligence in Medicine and Healthcare, Technical University of Munich, Munich, Germany; 3 Department of Computing, Imperial College, London, UK; 4 OpenMined, Oxford, UK

**Keywords:** Computer science, Software

## Abstract

The successful training of deep learning models for diagnostic deployment in medical imaging applications requires large volumes of data. Such data cannot be procured without consideration for patient privacy, mandated both by legal regulations and ethical requirements of the medical profession. *Differential privacy* (DP) enables the provision of information-theoretic privacy guarantees to patients and can be implemented in the setting of deep neural network training through the *differentially private stochastic gradient descent* (DP-SGD) algorithm. We here present *deepee*, a free-and-open-source framework for differentially private deep learning for use with the *PyTorch* deep learning framework. Our framework is based on parallelised execution of neural network operations to obtain and modify the per-sample gradients. The process is efficiently abstracted via a data structure maintaining shared memory references to neural network weights to maintain memory efficiency. We furthermore offer specialised data loading procedures and privacy budget accounting based on the *Gaussian Differential Privacy* framework, as well as automated modification of the user-supplied neural network architectures to ensure DP-conformity of its layers. We benchmark our framework’s computational performance against other open-source DP frameworks and evaluate its application on the *paediatric pneumonia dataset*, an image classification task and on the *Medical Segmentation Decathlon Liver* dataset in the task of medical image segmentation. We find that neural network training with rigorous privacy guarantees is possible while maintaining acceptable classification performance and excellent segmentation performance. Our framework compares favourably to related work with respect to memory consumption and computational performance. Our work presents an open-source software framework for differentially private deep learning, which we demonstrate in medical imaging analysis tasks. It serves to further the utilisation of privacy-enhancing techniques in medicine and beyond in order to assist researchers and practitioners in addressing the numerous outstanding challenges towards their widespread implementation.

## Introduction

Artificial Intelligence (AI) is a heavily data-centric domain: the success of machine learning (ML) models depends on the quality and quantity of data that is available during training. This is especially problematic in applications such as medical image analysis, in which high quality data is sparse and data utilisation is restricted. Medical data is highly sensitive, and regulatory, ethical and moral requirements restrict its sharing. These restrictions, although crucial, hinder the development of algorithms that generalise well and therefore prevent widespread deployment. Recent work^1^ finds that even algorithms approved for diagnostic use are often trained on small (i.e. less than 1000 cases), single centre datasets. Considering that state-of-the-art generic computer vision models are customarily trained on datasets such as ImageNet^2^ containing orders of magnitude more images, it becomes readily apparent that the access to more data will be strictly necessary for the development of the majority of deep learning applications in medical imaging to achieve the same success. Privacy-preserving machine learning is a nascent area of AI which proposes to bridge the gap between data utilisation and data protection through the application of privacy-enhancing techniques^3^. Among these, collaborative learning protocols such as federated learning have arguably witnessed the widest publicity^4^. They allow a confederation of clients to train ML models in a decentralised fashion and without sharing the raw data. However, a number of works suggest^5–7^ that on its own, federated learning is an insufficient measure of privacy preservation. In the setting of medical imaging, this can result in catastrophic privacy loss for affected patients. Prior work demonstrates that federated learning without additional privacy-enhancing techniques can be reverse-engineered to reconstruct high-fidelity images which encode diagnostic information about patients, such as the absence of a breast indicative of a prior history of breast cancer^8^. Moreover, three-dimensional medical imaging can be volumetrically rendered to reconstruct facial contours which enable patient re-identification^9^. Lastly, even when identifying attributes are not directly present in the image, the exploitation of side information by adversaries in the setting of linkage attacks, proven to represent a highly effective method for membership inference^10^, is also applicable to medical imaging databases given that large-scale public datasets of medical images are being assembled and—increasingly—publicly released. Thus, solutions based on information-theoretic privacy measures are required to provide comprehensive and quantifiable guarantees to the involved parties. Differential privacy (DP)^11^ has arisen as the gold standard in this regard. In brief, DP is the attribute of an algorithm to be approximately invariant to the inclusion or exclusion of individual patients, providing them with formal and quantifiable privacy guarantees. Although formally an information-theoretic privacy guarantee, in practice DP is typically achieved through *computationally secure* means, that is, an addition of carefully calibrated noise to the training process, making individual contributions indistinguishable from each other. In their seminal paper, Abadi et al.^12^ demonstrated the successful application of DP in the training of deep neural networks, termed *differentially private stochastic gradient descent* (DP-SGD). However, the authors of this and subsequent works noted that the utilisation of DP-SGD unavoidably negatively affects the utility of the resulting models, a well-known effect termed the *privacy-utility trade-off*^13^. Addressing this trade-off^14^ and ultimately enabling the widespread real-world utilisation of privacy-preserving ML in medical imaging and beyond requires the introduction of robust software tools, suitable for implementation within widely-used deep learning libraries and implementing current best practices.

We here present *deepee*, a software framework for differentially private deep learning based on the PyTorch^15^ machine learning library. Our main contributions can be summarised as follows:

- We present a technical implementation of the DP-SGD algorithm based on parallelised execution, which makes our framework universally compatible with any neural network layer while enabling substantial performance improvements.
- We implement state-of-the-art tools for production-level DP-SGD application including cryptographically secure random noise generation, automatic architecture modifications and privacy budgeting based on the *Gaussian Differential Privacy* (GDP) framework which offers a tight analysis of privacy consumed.
- We benchmark our toolkit against comparable DP-SGD implementations and analyse the behaviour of DP-SGD in the setting of two medical imaging deep learning tasks: classification and semantic segmentation
- Our framework is aimed at facilitating the application of DP-SGD to arbitrary data by non-experts. For this purpose, it exposes standardised application programming interfaces, is highly compatible with the *PyTorch* deep learning framework and automatically enforces the relevant details to ensure the formal correctness of the DP-SGD algorithm application.
- The source code of our framework is documented in detail, fully tested and available publicly and freely under a permissive, open-source license to enable easy maintenance, rapid detection and correction of potential security vulnerabilities and to encourage open-source contributions.

Two notable works have presented DP frameworks for the *PyTorch* machine learning library based on different technical implementations. The *Opacus* framework^16^ provides an implementation of the DP-SGD algorithm based on temporarily caching intermediate backpropagation results. This enables very high performance for specific deep neural network layer types. However, it does not ensure generic compatibility with any given neural network operation unless the procedure for obtaining said backpropagation results is explicitly defined on the user’s side. At the time of writing, the framework’s privacy analysis is still based on *Rényi DP* (RDP)^17^, whose guarantees are not as tight as Gaussian DP (GDP). The *Pyvacy*^18^ framework implements a generic version of DP-SGD based on serial execution. Despite its broad compatibility, this implementation is highly computationally inefficient, rendering it impractical for production-level use. The framework also lacks cryptographically secure random number generation and utility functions for automatic neural network architecture modification.

The *TensorFlow Privacy* framework^19^ and previous work based on the *JAX* machine learning framework^20^ share some characteristics of our library, such as utilisation of the GDP accounting technique or parallelisation, but they are based around different base libraries and thus are not directly comparable to our work.

## Results

### Technical overview

We begin by providing a brief technical overview of our framework. Implementation details can be found in the “Methods” section. In brief, *deepee* implements the DP-SGD algorithm in a memory-efficient and parallelised manner by increasing the efficiency of the *per-sample-gradient* calculation step drastically compared to serial processing. This occurs by creating one zero-memory-cost reference to the network’s weights for each sample in the minibatch, then performing a simultaneous (parallelised) forward and backward pass. This process introduces no additional assumptions about the network’s architecture and thus allows the application of the DP-SGD algorithm to any neural network architecture. This represents an improvement compared to prior work, which requires substantial user effort to manually specify the *per-sample* gradient calculations for unsupported layer types (e.g. *pixel shuffle or transposed convolutions, transformers, etc.*) or relies on performing forward and backward passes serially, thus magnifying time complexity. The framework furthermore is designed to guarantee the formal correctness of the DP-SGD procedure by e.g. removing Batch Normalisation layers from the architecture, employing cryptographically secure random noise and automatic privacy budgeting.

In the following, we demonstrate the utilisation of our framework in the settings of medical image classification and semantic segmentation. We present model performance in private and non-private settings to evaluate the expected privacy-utility trade-offs. Moreover, we compare our library’s computational performance with alternative implementations of the algorithm offered by the *Opacus* and *Pyvacy* frameworks.

### Chest radiography classification

The classification model achieved a mean receiver-operator characteristic area-under-the-curve (ROC-AUC) of 0.848 (range 0.814 to 0.881) in the private setting and of 0.960 (range 0.946 to 0.971) in the non-private setting (*DeLong*-test $p<0.001$, $N=10$). GDP accounting yielded a privacy budget ($\varepsilon$) of 0.52 at a noise multiplier of 3.0 and an $L_2$ clipping norm of 1.0, a tighter result than 0.62, which would have resulted from the utilisation of RDP analysis ($\delta =10^{-5}$). We observed that relaxing the privacy parameters (noise multiplier and clipping norm) resulted in a significant increase in classification performance of the private model (ROC-AUC in the relaxed privacy setting 0.882, range 0.868 to 0.899, *DeLong*-test vs. the strict privacy setting $p<0.001$, $N=10$) for an $\varepsilon$ of 2.69 (GDP accounting) or 2.81 (RDP accounting). Even in the relaxed setting however, the model still significantly underperformed compared to non-private training (*DeLong*-test vs. non-private training $p<0.001$, $N=10$). These results are summarised in Table 1.

**Table 1:** Classification performance (measured as mean receiver-operator characteristic area-under-the-curve (ROC-AUC)) on the paediatric chest radiography binary classification dataset.

Model	ROC-AUC	GDP $\varepsilon$	RDP $\varepsilon$
Non-private	0.960 [0.946 to 0.971]	$\infty$	$\infty$
Private	0.848 [0.814 to 0.881]	0.52	0.64
Private (relaxed)	0.882 [0.868 to 0.899]	2.69	2.81

Ranges in angled brackets. The non-private model significantly outperformed the private model in both the high-privacy setting and the relaxed privacy setting, while the private model trained with relaxed privacy guarantees significantly outperformed the private model with strict guarantees.

### Semantic segmentation of computed tomography images

In the semantic liver tissue segmentation task, the non-privately and privately trained models produced nearly identical results: The mean Dice coefficient achieved by the privately and the non-privately trained models was 0.943 (range 0.941 to 0.945), and 0.950 (range 0.948 to 0.951, N = 5), respectively. This segmentation performance of the privately trained model was attained at an $\varepsilon$ of 0.12 (GDP) or 0.35 (RDP) and a $\delta$-value of $10^{-5}$, resulting from a noise multiplier of 5.0 and an $L_2$ clipping norm of 0.5, indicating that the provision of strict privacy guarantees was possible in this setting without a notable trade-off in model performance. Results are summarised in Table 2.

**Table 2:** Segmentation performance (measured by the mean Dice coefficient) on the liver semantic segmentation dataset.

Model	Dice coefficient	GDP $\varepsilon$	RDP $\varepsilon$
Non-private	0.950 [0.948 to 0.951]	$\infty$	$\infty$
Private	0.943 [0.941 to 0.945]	0.12	0.35

Ranges in angled brackets. The privately trained and the non-privately trained models performed on par despite the provision of stringent privacy guarantees in the privately trained setting.

### Computational performance comparison

Table 3 presents a comparison of the computational performance and memory consumption of our framework versus the *Opacus* and *Pyvacy* libraries in the classification and segmentation settings. We found our framework to offer significantly faster computational performance in the segmentation setting compared to *Opacus* (*Student’s* t-test $p<0.001$) and *Pyvacy* ($p<0.001$). *Opacus* significantly outperformed our framework ($p<0.001$) and *Pyvacy* ($p<0.001$) in the classification task. (All 25 batches of 32 examples over N = 5 repetitions).

Our framework required significantly less memory than *Opacus* in both the classification and segmentation setting (*Student’s* t-test $p<0.001$). *Pyvacy*, due to serial processing of the individual samples in each minibatch suffers from a drastically diminished computational performance, however requires significantly less memory than both other frameworks as a result of only needing to cache a single sample’s gradients at a time (*Student’s* t-test $p<0.001$, all N = 6 repetitions).

Moreover, to exemplify our framework’s compatibility, we benchmarked an additional U-Net architecture utilising transposed convolutions as described in the original work^21^. The *Opacus* framework is incompatible with transposed convolutions and could thus not be assessed. *Pyvacy*, while requiring less memory ($p<0.001$), again was significantly slower per batch compared to *deepee* ($p<0.001$).

**Table 3:** Computational performance (median time for N = 25 batches of 32 examples in seconds over N = 5 repetitions) and mean peak memory consumption (one batch of 32 examples in MiB, N = 6 repetitions) of the compared frameworks for the classification and segmentation benchmarks.

Task	*deepee* (ours)	*Opacus*	*Pyvacy*
Classification	38.82 s [38.67 to 39.08]	16.39 s [16.29 to 16.69]	73.11 s [72.41 to 75.40]
	6366 MiB [6201 to 6448]	7014 MiB [6816 to 7213]	2044 MiB [1992 to 2102]
Segmentation	70.89 s [70.41 to 71.01]	78.47 s [78.08 to 79.86]	97.89 s [97.26 to 99.16]
	9770 MiB [9508 to 9829]	9909 MiB [9812 to 10112]	2085 MiB [1890 to 2205]
Segmentation (Transposed Conv.)	47.27 s [45.12 to 51.15]	–	64.68 s [62.76 to 66.32]
	12014 MiB [11598 to 12249]	–	1537 MiB [1399 to 1620]

Ranges in angled brackets. The Segmentation (Transposed Conv.) row showcases framework performance in a U-Net architecture using transposed convolutions. *Opacus* is incompatible with this layer type.

## Discussion

Here we present a novel technical implementation of the DP-SGD algorithm which we demonstrate and benchmark in the setting of medical image analysis. We found our technique’s computational performance and memory consumption to be comparable to state-of-the-art frameworks without a requirement for user-side modifications. Our framework thus provides formal privacy guarantees regardless of the dataset, learning task and of model selection. Moreover, by leveraging the current state-of-the-art in DP analysis, we demonstrate tighter privacy bounds compared to previous DP accounting techniques. The two applications presented provide evidence for the usefulness of our DP-SGD algorithm in real-world medical image processing.

Medical imaging represents a domain in which privacy-utility trade-offs are especially problematic, as models that generalise well require large and diverse multi-centre datasets during training and must not divulge personal test data once deployed. Such demands are—for example—placed on ML models utilised for remote diagnosis-as-a-service^22^, where expert-level algorithm performance is expected, while the model may be exposed to probing by malicious third parties. Formal security and secrecy mechanisms such as model encryption can only partially address this requirement, as even encrypted models have been found to leak sensitive information in previous work^23,24^. Similarly, distributed learning techniques such as federated learning, often touted as being “privacy-preserving” because the data does not leave its owner, have been proven ineffective against attackers who participate in the training protocol and are able to capture updates submitted by other participants^5,6^. Differentially private model training therefore stands as the only formal mechanism for privacy protection, able to shield models from feature reconstruction, model inversion and membership inference attacks^6,25^. Moreover, recent work demonstrates that DP can reduce the susceptibility of models to other adversarial interference such as *back-door attacks*^26^, which can be attributed to the increased robustness of DP models imparted through the regularising properties of noise addition^27^.

Inherent to these beneficial properties of DP model training is—however—also an unavoidable net reduction in model utility. We identify three key components of this utility penalty: (1) Diminished task-specific performance, e.g. in classification or segmentation tasks; (2) computational performance penalties through an increase in training time and memory consumption and (3) incompatibilities of the DP-SGD algorithm with the neural network architecture. Our work attempts to address all three of these points.

The use-cases chosen in our study, image classification and segmentation, represent two typical workflows in medical imaging analysis. Interestingly, we observed a marked performance decrease in the private classification task compared to non-private model training even under relaxed privacy guarantees. Semantic segmentation was possible under very strong privacy notions with unexpectedly strong performance. The only other work to report an $\varepsilon$-value in a medical image segmentation task^28^ utilises a different DP technique, whose utilisation results in a high privacy expenditure of over 120 under the study’s assumptions, compared to 0.12 in our work. No previous work—to our knowledge—reports $\varepsilon$-values for medical image classification. At present, it is not yet conclusively investigated to which extent the difficulty of the task, the choice of model and the specific training technique influence the privacy-utility trade-off. Future work will thus have to elucidate these relationships and expand on recent studies in this direction^13,14,29^.

Besides these factors, more refined techniques for privacy accounting are able to offer an improved analysis of the DP mechanism and thus allow higher utility. In the medical imaging domain, the combination of high utility and low privacy budget is particularly important. As datasets are complex, highly sensitive and typically small, each individual in the dataset experiences a relatively higher privacy loss. A tight privacy analysis allows training the models for a longer time before the privacy budget is exhausted, enabling higher task-specific performance and therefore, a better diagnostic prediction. Our work utilises Gaussian Differential Privacy, a recently introduced DP formulation which—through a tight characterisation of the sub-sampled Gaussian noise mechanism utilised in DP-SGD—improves the outlook on the spent privacy budget compared to previous frameworks. It is expected that further advances, such as individual privacy accounting^30,31^ will increase the granularity of privacy tracking further, allowing for the preservation of even higher utility during algorithm training.

Our main technical contribution is the introduction of a parallelised execution model for the DP-SGD algorithm within the *PyTorch* framework, which enables both fast performance and efficient memory utilisation. In addition, our technique-contrary to frameworks relying on the *a priori* specification of *per-sample* gradient calculations such as *Opacus*- is compatible by default with *any* neural network operation including (but not limited to) transformer architectures or transposed convolutions, as seen above. This disparity is discussed in^20^, a line of work complementary to ours, whose authors utilise *just-in-time* compilation and vectorised execution to increase DP-SGD performance, albeit within a different machine learning framework. We moreover see a target for future work focused around automatic differentiation with inbuilt support for obtaining and manipulating *per-sample* gradients. After all, the requirement to calculate *per-sample* gradients in current DP-SGD frameworks stems from the inherent design philosophy of reverse-mode automatic differentiation systems, which are focused on efficiently obtaining gradients for minibatches but not for individual samples. We moreover note that techniques concerned with approximate gradient calculations^32^ have some overlap with the objectives of DP-SGD, which inherently performs an “imprecise” gradient update step through noise addition, and could thus be utilised for increased performance, after considering their effect on privacy guarantees.

Similar to previous work^16^, our work offers the capability to automatically modify the neural network architecture in case layers incompatible with DP-SGD are included. An example of this phenomenon in the current work is the deactivation of running statistics collection for Batch Normalisation layers. Moreover, our framework includes support for cryptographically secure random noise generation which is crucial to avoid vulnerabilities associated with default pseudo-random number generators^33^.

We consider some limitations of our work: Our framework’s focus is to provide a generic framework for DP-SGD and the examples presented represent a simplification of real-life use-cases intended to illustrate its utilisation in medical imaging. In the segmentation case-study in particular, we provide image-level privacy guarantees, whereas a real-life deployment would be adjusted to offer patient-level guarantees (that is, a “summary” of privacy guarantees derived from the utilisation of all images of a single patient). Moreover, DP techniques purpose-designed for high performance in classification, such as PATE^34^ could yield improved privacy-utility trade-offs in the classification use-case compared to DP-SGD, however at the cost of not generalising well to other tasks such as segmentation^28^ and an additional assumption of a publicly available dataset that cannot be reliably expected in a sensitive setting, such as medical imaging.

In conclusion, our work aims to facilitate the utilisation of differentially private deep learning in everyday practice. It is well-suited to privacy-sensitive tasks such as medical imaging analysis. We publicly release our framework and experiments in the hope that it will stimulate future research and lead to the design of improved algorithms and training techniques to enable privacy-preserving machine learning with improved algorithm utility in medical imaging and beyond.

## Methods

### Framework implementation details

#### User-facing components

Our framework provides the following high-level user-facing components: (1) A collection of procedures to automatically modify the neural network architecture in case it contains layers which are incompatible for utilisation with DP-SGD. One example is the Batch Normalisation layer which maintains a (non-private) running average of statistics over more than one training example and is thus not compatible with the notion of *per-sample* gradient calculations, which are required in DP-SGD. (2) A data structure encapsulating the user-supplied model architecture, responsible for the main model training and evaluation loop. This *wrapper* internally maintains one copy of the user-supplied model per sample in the minibatch, performs a parallelised forward and backward pass over the minibatch and abstracts the gradient clipping and noise application of the DP-SGD procedure. (3) A *privacy accounting* mechanism for keeping track of the privacy spent at each training step and including a procedure to automatically interrupt the training if the privacy budget is exhausted. The system is supplemented by a cryptographically secure random number generator^35^ suitable for use on the graphics processing unit and capable of parallelising the random noise generation step of the DP-SGD algorithm.

#### DP-SGD algorithm implementation

We implement the DP-SGD algorithm as described in^12^. In brief, the algorithm consists of the following steps:

1. Performing a forward pass on a minibatch of samples
2. Calculating the gradient of the loss with respect to each sample individually (*per-sample gradients*)
3. Normalising (*clipping*) the per-sample gradients to a predefined $L_2$-norm
4. Aggregating the per-sample gradients by averaging or summing over the minibatch axis
5. Adding calibrated Gaussian noise to the resulting gradient vector

In practice, step (2) of the above-mentioned procedure is the most time-consuming subroutine of the algorithm, as automatic differentiation systems are not designed with per-sample gradient computation in mind. To tackle this problem, our framework first creates a copy of the neural network for each sample in the minibatch and then performs step (1) of the algorithm above in parallel by dispatching one execution thread per minibatch sample. Thus, the backpropagation procedure yields per-sample gradients per definition (step (2) above). This approach has several benefits: It is computationally efficient as it is performed in parallel over the minibatch leveraging multi-threaded execution on e.g. the graphics processing unit (GPU). Moreover, memory only needs be allocated once for the neural network weights (as all copies share the same weights). Lastly, the process is entirely generic and can be used for any arbitrary neural network architecture without the requirement for user interaction. A similar technique to ours, albeit based on serial execution instead of a parallelised forward pass and only demonstrated for convolutional neural networks, is presented in^36^, reportedly going back to (unpublished) work by Goodfellow et al.

### Datasets

#### Classification task

We evaluated our framework on a classification task on chest radiographs from the Paediatric Pneumonia dataset originally described in^37^. Originally, the task was formulated as three-class classification, however we merged the *viral* and *bacterial* pneumonia labels to obtain a binary classification task, in which the algorithm attempts to predict whether the radiograph shows signs of pneumonia or not. The dataset contains 1339 training images of healthy patients and 3824 images of patients that present evidence of pneumonia. The dataset is pre-split into a training (n = 5163) and a test set (n = 624). We further split the training set into $85\%$ training data (n = 4389) and $15\%$ validation data (n = 774). To account for class imbalance, we weighted the resulting loss by one minus the proportion of the dataset of the class. Data augmentation was performed using affine transformations (rotation, scaling, translation, shearing). Every occurence of an image from the same patient, regardless whether it was augmented or not, was counted against the total privacy expenditure. We trained the models for 20 epochs using the Adam optimiser in the non-private setting and the Stochastic Gradient Descent (SGD) optimiser in the private setting. Learning rates were determined using a learning rate finding algorithm^38^ and set to 0.005 in both settings. Learning rate scheduling with halving of the learning rate on stagnation of the validation loss for two consecutive epochs was employed.

#### Semantic segmentation task

For the semantic segmentation task, we used the Medical Segmentation Decathlon (MSD) Liver segmentation dataset^39^. We split the available data into a training set (n = 5184), a validation set (n = 640) and a held-out test set (n = 2560), mindful to enforce strict patient independence between the training/validation sets and the test set. The task was re-formulated as a binary segmentation task, in which the liver tissue pixels (including tumours) are labelled as 1 and the background as 0. For augmentation purposes, affine transformations (rotation, translation, scaling, flipping) alongside random Gaussian noise were applied to the input images. Every occurence of an image from the same patient, regardless whether it was augmented or not, was counted against the total privacy expenditure. The model was trained for 20 epochs in the non-private setting. In the private setting, we limited the number of epochs to 5 in order to maintain a low privacy budget. Learning rates were determined using the same learning rate finding algorithm and set to 0.01, while utilising the Adam optimiser in both cases. Learning rate scheduling was performed in the same manner as for the classification task.

### Model training

For the classification task, we utilised the same model architecture in the private and non-private setting, namely a VGG-11^40^ architecture with Batch Normalisation. However, in order to satisfy the assumptions essential for DP training, the collection of running statistics of Batch Normalisation layers was disabled for both non-private and DP training. For the segmentation task, we use a modified U-Net architecture^21^ utilising VGG-11 with Batch Normalisation as a backbone^41^. Similarly to the classification task, the running statistics collection was disabled. The $\delta$-parameter was set to $10^{-5}$ in all cases.

### Computational performance and memory benchmarks

For the purposes of computational performance benchmarking we measured the time to train for 25 steps with a minibatch size of 32 on the tasks we presented above, i.e., binary classification on 224x224 sized images and the segmentation of 256x256 images. Each measurement was repeated five times.

For memory utilisation benchmarking, a minibatch size of 32 images at a resolution of $256 \times 256$ was used, with a single channel for the classification benchmark and three channels for the segmentation benchmark. All benchmarks were conducted in triplicate to ensure stability between runs and repeated on two operating systems, *macOS 11.2.3* and *GNU Linux* on the 5.4.0-72 kernel (total N = 6 runs). Peak memory consumption was measured using the *Python* programming language (*CPython* v. 3.8.8) standard library module *resource*.

### Statistical methods

Areas under the ROC-curve were compared using the *DeLong*-test as described in^42^. Continuous variables were compared using the *Student’s* t-test. *Bonferroni’s* correction was used for three-way comparisons with the adjusted statistical significance threshold set to $p=0.016$.

## Data Availability

The *deepee* framework and code to reproduce the experiments is available at https://github.com/gkaissis/deepee. The paediatric pneumonia dataset is available from https://data.mendeley.com/datasets/rscbjbr9sj/3. The liver segmentation dataset is available from http://medicaldecathlon.com.
